# The ppm Operon Is Essential for Acylation and Glycosylation of Lipoproteins in *Corynebacterium glutamicum*

**DOI:** 10.1371/journal.pone.0046225

**Published:** 2012-09-28

**Authors:** Niloofar Mohiman, Manuela Argentini, Sarah M. Batt, David Cornu, Muriel Masi, Lothar Eggeling, Gurdyal Besra, Nicolas Bayan

**Affiliations:** 1 Institut de Biochimie et de Biophysique Moléculaire et Cellulaire, Université Paris-Sud, Orsay, France; 2 Centre National de la Recherche Scientifique UMR 8619, Orsay, France; 3 Centre National de la Recherche Scientifique, Institut de Chimie des Substances Naturelles, Gif sur Yvette, France; 4 School of Biosciences, University of Birmingham, Birmingham, United Kingdom; 5 Forschungszentrum Juelich GmbH, Jülich, Germany; Hopital Raymond Poincare - Universite Versailles St. Quentin, France

## Abstract

**Background:**

Due to their contribution to bacterial virulence, lipoproteins and members of the lipoprotein biogenesis pathway represent potent drug targets. Following translocation across the inner membrane, lipoprotein precursors are acylated by lipoprotein diacylglycerol transferase (Lgt), cleaved off their signal peptides by lipoprotein signal peptidase (Lsp) and, in Gram-negative bacteria, further triacylated by lipoprotein N-acyl transferase (Lnt). The existence of an active apolipoprotein N-acyltransferase (Ms-Ppm2) involved in the N-acylation of LppX was recently reported in *M. smegmatis*. Ms-Ppm2 is part of the ppm operon in which Ppm1, a polyprenol-monophosphomannose synthase, has been shown to be essential in lipoglycans synthesis but whose function in lipoprotein biosynthesis is completely unknown.

**Results:**

In order to clarify the role of the ppm operon in lipoprotein biosynthesis, we investigated the post-translational modifications of two model lipoproteins (AmyE and LppX) in *C. glutamicum* Δ*ppm1* and Δ*ppm2* mutants. Our results show that both proteins are anchored into the membrane and that their N-termini are N-acylated by Cg-Ppm2. The acylated N-terminal peptide of LppX was also found to be modified by hexose moieties. This *O-*glycosylation is localized in the N-terminal peptide of LppX and disappeared in the Δ*ppm1* mutant. While compromised in the absence of Cg-Ppm2, LppX *O-*glycosylation could be restored when Cg-Ppm1, Cg-Ppm2 or the homologous Mt-Ppm1 of *M. tuberculosis* was overexpressed.

**Conclusion:**

Together, these results show for the first time that Cg-Ppm1 (Ppm synthase) and Cg-Ppm2 (Lnt) operate in a common biosynthetic pathway in which lipoprotein N-acylation and glycosylation are tightly coupled.

## Introduction

Bacterial lipoproteins are a subgroup of exported proteins anchored into the membrane surface by two or three acyl chains covalently linked to their conserved N-terminal cysteine. They represent a functionally heterogeneous family of proteins involved in several functions ranging from cellular physiology to cell division or virulence in pathogenic species [1]. In Gram-negative bacteria, they are involved in crucial steps of cell envelope biogenesis such as outer membrane protein insertion by the Bam machinery [2] or peptidoglycan assembly with penicillin binding protein machineries [3], [4]. In Gram-positive bacteria, they often act as substrate-binding proteins (SBP) associated with ATP binding cassette (ABC) transporters [5].

The genome of *Mycobacterium tuberculosis* H37Rv encodes about 100 putative lipoproteins among which half of them are specific to the genus *Mycobacterium* and have no assigned function [6]. Functional data on the involvement of mycobacterial lipoproteins in ABC transporters (PhoS123), two component regulatory systems (LpqB and LprF) [7], [8], biosynthetic pathways of cell wall components (LpqW, LppX) [9], [10], resuscitation of dormant cells (RpfB) [11], cytokine responses and regulation of antigen presentation by host cells as Toll like-receptor ligands (LprA, LprG, LpqH) have been reported [12], [13]. Although very few of them have been extensively characterized biochemically, it is striking to note that most lipoproteins studied so far are also glycosylated [14] as it was shown for PstS, a lipoprotein in the related actinomycete *Streptomyces coelicolor* [15] This original feature has been very clearly shown for LpqH [16], LprF [17], SodC [18] and PhoS1 [19]. The role of lipoprotein glycosylation is not yet clear, but could be a common post-translational modification that would act as a signal for protein export to the cell wall or the extracellular medium. Alternatively, it has been suggested that glycosylation might protect surface-exposed lipoproteins from proteolytic degradation [16], [18]. Speculatively, glycosylation of mycobacterial lipoproteins might represent a signal to induce or to avoid a “shaving” mechanism similar to that proposed by Tjalsma [20] for the release of *Bacillus subtilis* lipoproteins in the culture medium.

Lipoproteins are synthesized as prolipoproteins and translocated across the plasma membrane via the Sec or TAT machinery. Subsequent post-translational modifications take place on the outer leaflet of the plasma membrane [21]. Typically, the acceptor residue for lipid modification is a conserved cysteine included in a consensus motif known as the lipobox (L(A,V)_-4_-L_-3_-A(S)_-2_-G(A)_-1_-C_+1_). In the first step, the cysteine (C_+1_) residue that will become the N-terminal amino acid of the mature lipoprotein receives a diacylglyceride at its sulfhydryl group through the action of the phosphatidylglycerol::prolipoprotein diacylglyceryl transferase (Lgt). Then, the N-terminal signal peptide is processed by prolipoprotein signal peptidase A (LspA), resulting in an apolipoprotein. The third and last step is the acylation of the N-terminal cysteine residue by apolipoprotein *N*-acyl transferase (Lnt), generating a mature triacylated lipoprotein. This last step is conserved throughout Gram-negative bacteria, and it was shown to be a prerequisite for lipoprotein sorting to the outer membrane by the Lol system [22]. Although recent results showed that lipoproteins are also triacylated in low % G+C Gram-positive bacteria [23], [24], [25], [26], Lnt is only found in high % G+C Gram-positive bacteria such as *Mycobacterium or Streptomyces*. Tschumi *et al.* [27] clearly showed that LppX, a lipoprotein of *M. tuberculosis* essential for Phthiocerol dimycocerosates (DIM) exposure at the cell surface of *M. tuberculosis*, is triacylated in *M. smegmatis* by the MSMEG_3860 gene product. This protein was formerly annotated as Ms-Ppm2 because it interacts with Ms-Ppm1, a polyprenol-monophosphomannose (PPM) synthase essential for lipomannan (LM) and lipoarabinomannan (LAM) biosynthesis [28], [29]. In *M. smegmatis*, Ms-*ppm2* is located upstream of Ms-*ppm1* within the ppm operon, while *M. tuberculosis* Rv2051c (Mt-*ppm1*) encodes a bi-domain protein in which the N-(D1) and C-terminal (D2) domains carry Lnt and PPM synthase activities, respectively [30]. This strong genetic link suggests that the *ppm1* and *ppm2* gene products are involved in a common biosynthetic pathway. However, functional connection(s) between lipoprotein and lipoglycan biogenesis pathways are unknown. By analogy with the mammalian dolichol-phosphomannose synthase, it has been proposed that *M. smegmatis*Ms-Ppm1 interacts with an inner membrane protein and that this interaction is required for stability and activity [29]. Consistent with this idea, Ms-Ppm2 has been shown to interact with Ms-Ppm1 in a bacterial two-hybrid assay [29]. The function of Ms-Ppm1 in lipoprotein biosynthesis has never been tested in *M. smegmatis* since the corresponding mutant is not viable. *Corynebacterium glutamicum* is a non-pathogenic bacterium widely accepted as a useful model to depict the cell wall biogenesis of *Corynebacterineae* including *M. tuberculosis*. The genetic organization of the *ppm* genes is conserved between *M. smegmatis* and *C. glutamicum*. Mutants in Cg-*ppm1* (Δ*ppm1*) or Cg-*ppm2 (*Δ*ppm2*) have been generated previously and are viable [28]. The Δ*ppm1*mutant is highly affected in growth and do not synthetize any lipoglycans, LM or LAM. In contrast, the Δ*ppm2* mutant although affected in LM and LAM biosynthesis is not impaired for growth (our unpublished data and [28]). In this work, we investigated the function of Cg-Ppm1, Cg-Ppm2 and their putative interplay in lipoprotein biogenesis. We analyzed the acylation and the glycosylation status of two model lipoproteins AmyE [31], an abundant lipoprotein of *C. glutamicum* (a putative sugar substrate binding protein) and LppX of *M. tuberculosis* [9], [14], [27] in various strains affected in *ppm1* or *ppm2*.

## Results

### AmyE is a Bona Fide Lipoprotein of *C. glutamicum*

According to predictions based on bioinformatics tools, the genome of *C. glutamicum* strain ATCC 13032 encodes 84 putative lipoproteins, representing about 2.8% of its total proteome (DOLOP [32]). In *C. glutamicum*, as in other Actinomycetes, these include many SBP of ABC transporters. Among these, AmyE (NCgl2375) is annotated as a putative maltose-binding protein and has been detected as a major spot in the proteome of *C. glutamicum*, suggesting it is well expressed in standard laboratory conditions [31]. We chose AmyE as a model to study the biogenesis of lipoproteins in *C. glutamicum*. The DNA sequence corresponding to the ORF of *amyE* including its putative promoter was amplified and cloned in an *E. coli/C. glutamicum* shuttle vector pCGL482 in frame with a 3′ sequence encoding a 6xHis tag (AmyE). A variant of *amyE*, with a point mutation converting the cysteine +1 of the lipobox into a leucine (AmyE^C1L^), was obtained by site directed mutagenesis and used as a control in all studies.

**Figure 1:**
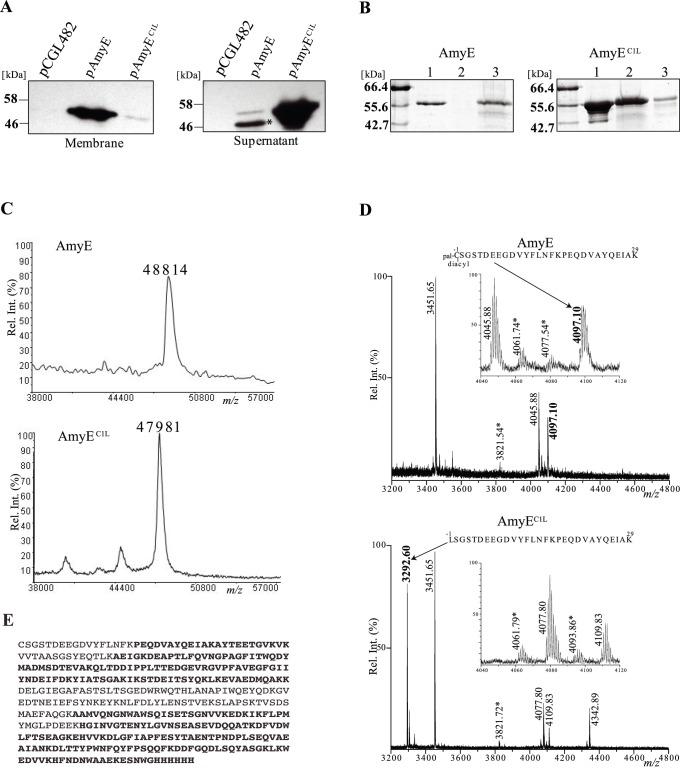
Lipoprotein AmyE is triacylated. **A.** AmyE localization in *C. glutamicum* wild-type cells expressing AmyE (pAmyE) or a variant of AmyE with a point mutation substituting the cysteine +1 by a leucine (pAmyE^C1L^). An empty vector (pCGL482) was used as a control. Membrane and secreted proteins were analyzed by SDS-PAGE followed by immunoblotting using monoclonal anti-his antibodies. The band labeled with an asterisk corresponds to a shorter form of AmyE. **B.** AmyE (left panel) and AmyE^C1L^ (right panel) were purified and analyzed by SDS PAGE before (lane 1) or after Triton X114 extraction. Proteins from both aqueous (lane 2) and detergent (lane 3) phases were precipitated and loaded on the gel. **C.** MALDI mass measurements of intact purified AmyE and AmyE^C1L^ proteins. Estimated mass accuracy is 150 Da. **D.** MALDI PMFs of AmyE and AmyE^C1L^ proteins purified from *C. glutamicum* wild-type strain. The *m/z* 3200–4800 region of the mass spectra of AmyE and AmyE^C1L^ tryptic peptides after DDM/CHCl_3_-CH_3_OH treatment is shown and significant monoisotopic [M+H]^+1^ peaks are indicated. Upper panel: *m/z* 4097.10 (bold) corresponds to the triacylated AmyE_1–29_ peptide while *m/z* 3451.65, 3821.53 and 4045.88 match to internal tryptic peptides, AmyE_334–365_, AmyE_366–397_ and AmyE_55–91_ respectively. *m/z* 4061.74 and 4077.54 peaks could correspond to mono- and di- oxidized AmyE_55–91_ peptides. Bottom panel: *m/z* 3292.60 (bold) corresponds to the C1L-mutated AmyE_1–29_ peptide while *m/z* 3451.65 and 3821.72 match to AmyE_334–365_ and AmyE_366–397_ peptides. The *m/z* 4061.79, 4077.80, 4093.86 and 4109.83 peaks match to mono-, di-, tri- and tetra-oxidized AmyE_55–91_ peptides. The *m/z* 4342.89 peak corresponds to the di-oxidized AmyE_277–315_ peptide. It’s worth noting that AmyE^C1L^ peptides were more often detected in the oxidized state than AmyE peptides. Insets aim at emphasizing the specificity of the *m/z* 4097.10 signal detected only in the AmyE spectrum. Asterisks indicate that *m/z* assignments are not accurate because of low mass resolution and weak signal/noise ratio. **E.** Sequence of the recombinant purified wild-type AmyE protein. Identified unmodified peptides after trypsin digestion and DDM/CHCl_3_-CH_3_OH treatment are shown in boldface type on the amino acid sequence of recombinant AmyE.

Plasmids expressing AmyE and AmyE^C1L^ were transformed in *C. glutamicum* ATCC 13032. To examine the fate of AmyE proteins, cells were grown overnight, membranes and culture supernatants were prepared and analyzed by SDS-PAGE and immunoblotting (Fig. 1A). AmyE is detected as a 55 kDa polypeptide mostly present in the membrane fraction. Its apparent molecular weight on SDS-PAGE is slightly higher than the calculated molecular weight of the mature AmyE (47895 Da). Small amounts of a shorter form were found secreted in the culture medium and may have been released by proteolysis. Such a truncated form has also been detected previously by Hermann *et al.* [31]. In contrast, AmyE^C1L^ is highly expressed but only detected in the culture supernatant as a 55 kDa species (Fig. 1A). To gain some insight on AmyE modifications, both variant forms were purified by affinity chromatography either from the membrane fraction (AmyE) or the culture supernantant (AmyE^C1L^). First, both proteins were subjected to SDS-PAGE before or after Triton X114 extraction. While both forms have a similar Rf on SDS-PAGE, AmyE is recovered in the detergent phase and AmyE^C1L^ remains in the soluble fraction (Fig. 1B). This is an indication that AmyE, but not AmyE^C1L^, has a hydrophobic moiety probably corresponding to acylation of its cysteine +1 (C+1). Second, purified AmyE and AmyE^C1L^ were subjected to protein sequence analysis. Edman degradation of AmyE^C1L^ resulted in an N-terminal sequence starting at the modified leucine +1 residue (LSGSTD) while no sequence could be obtained from AmyE. These results indicate that the mutant protein has been cleaved off its signal peptide and that AmyE carries a modification on its N-terminal amino group blocking its degradation by Edman reagents. To further characterize this modification, purified intact AmyE and AmyE^C1L^were analyzed by MALDI. A significant difference of 918 Da between the expected and the measured mass of AmyE (M_expected_ = 47896 Da and M_measured_ = 48814±150 Da) was observed, while there was no significant difference between the expected and the experimental measurement of AmyE^C1L^ protein (M_expected_ = 47896 and M_measured_ = 47981±150 Da) (Fig. 1C). The 918 Da mass difference is consistent with a putative triacylation of AmyE. In order to characterize the post-translational modification of AmyE more precisely, MALDI peptide mass fingerprinting analyses (PMF) were performed after in-gel trypsin digestion followed by peptide extraction with dodecylmaltoside (DDM) and chloroform/methanol. This procedure, describedby Ujihara *et al.* [33], allowed the efficient detection of acylated peptides. AmyE and AmyE^C1L^ peptide profiles were very similar except for two main peaks shown in Fig. 1D. The *m/z* 4097.10 peak was detected exclusively in the AmyE spectrum while the *m/z* 3292.60 peak was only associated to the AmyE^C1L^ spectrum. The latter signal matches to the unmodified N-terminal peptide of AmyE^C1L^ (mass accuracy = 14 ppm), while the former most likely corresponds to the N-terminal peptide of AmyE harboring a post-translational modification of 814 Da (estimated mass accuracy <20 ppm). This is consistent with triacylation of AmyE as the 814 Da modification might correspond to the addition of a diacylglyceryl (576 Da) and a palmitoyl moiety (238 Da). Additional peaks that are apparently distinctive between the spectra of AmyE and AmyEC1L are attributable to differential oxidation of internal tryptic peptides of AmyEC1L compared to AmyE, as detailed in the Legend to Fig. 1.

### Cg-ppm2 is Essential for N-palmitoylation of AmyE

**Figure 2:**
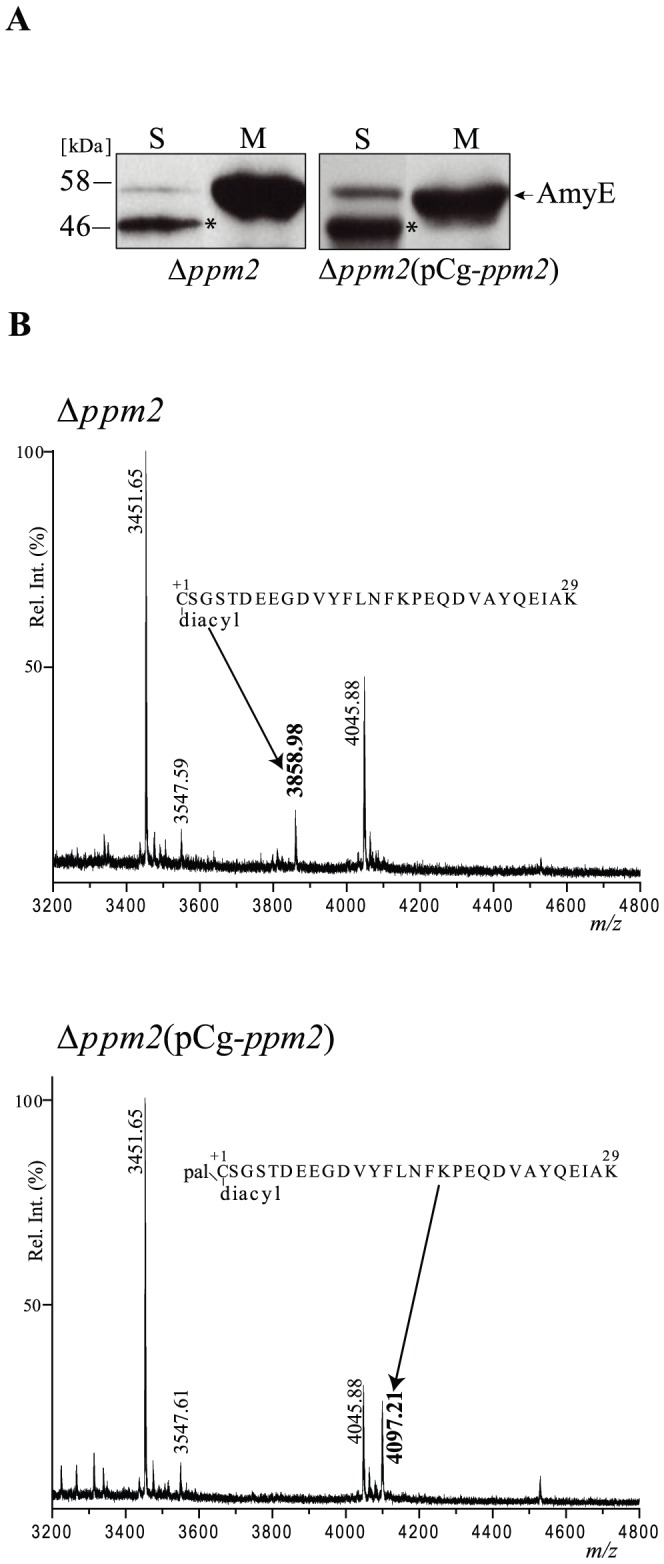
Cg-Ppm2 exhibits apolipoprotein N-acyltransferase activity. A. Localization of AmyE in *C. glutamicum* Δ*ppm2* or Δ*ppm2* (Cg-*ppm2*) strains. Membrane (M) and secreted (S) proteins were analyzed by SDS-PAGE followed by immunoblotting using monoclonal anti-his antibodies. The band labeled with an asterisk corresponds to a shorter form of AmyE. **B.** MALDI PMFs of AmyE protein purified from ΔCg-*ppm2* and ΔCg-*ppm2* (Cg-*ppm2*) strains. The *m/z* 3200–4800 region of the mass spectra of AmyE tryptic peptides after DDM/CHCl_3_-CH_3_OH treatment is shown and significant monoisotopic [M+H]^+1^ peaks are annotated. Upper panel: *m/z* 3858.98 (bold) corresponds to the diacylated AmyE_1–29_ peptide while *m/z* 3451.65, 3547.59 and 4045.88 match to internal tryptic peptides, AmyE_334–365,_ AmyE_60–91_ and AmyE_55–91,_ respectively. Bottom panel: *m/z* 4097.21 (bold) corresponds to the triacylated AmyE_1–29_ peptide while *m/z* 3451.65, 3547.61 and 4045.88 match to AmyE_334–365,_ AmyE_60–91_ and AmyE_55–91_ peptides.

In Gram-negative bacteria, lipoproteins are successively acylated by Lgt (formation of a thioester linkage between the conserved C+1 and a diacylglycerol) and Lnt (aminoacylation of the N-terminal C+1) to form mature triacylated proteins. While the Lgt-mediated reaction is universally conserved in bacteria [1], N-acylation was thought to be restricted to Gram-negative bacteria. Yet, genomic analysis revealed the presence of Lnt homologs in high % G+C Gram-positive bacteria, such as Actinomycetes including Mycobacteria and Corynebacteria. More recently, Sander and collaborators [27] convincingly showed that an Lnt homolog (MSMEG_3860 or Ms*-*Ppm2) was fully active in *M. smegmatis* and was able to N-acylate mycobacterial lipoproteins such as LppX and LprF. The homolog of Ms*-ppm2* was observed in the genome of *C. glutamicum* and corresponds to *NCgl1424* formerly known as Cg-*ppm2.* This gene is putatively co-transcribed with Cg-*ppm1*, which encodes a polyprenol monophosphomannose synthase involved in lipoglycan synthesis [28]. However, the putative role of Cg-Ppm2 in N-acylation of lipoproteins in *C. glutamicum* has never been tested. AmyE was expressed in a Δ*ppm2* mutant and in a complemented strain in which Cg-*ppm2* was reintroduced *in trans*on a plasmid under *tac* promoter control (pCg*-ppm2*). In both cases, AmyE was mainly found in the membrane fraction, similar to that observed in the wild-type strain (Fig. 2A and Fig. 1A). AmyE proteins from the membrane fraction of Δ*ppm2* or the complemented strains (Δ*ppm2*-pCg-*ppm2*) were purified to homogeneity and analyzed by MALDI PMF using the same procedure as described above. Notably, the *m/z* 4097.10 peak detected for AmyE purified from the wt strain (Fig. 1D and Fig. 2B) was absent from the spectrum of the AmyE purified from the Δ*ppm2*mutant, but was detected again in the spectrum of AmyE purified from the complemented strain (Fig. 2B). Instead, in the Δ*ppm2*mutant, we identifieda new *m/z* 3858.98 peak. This signal most likely corresponds to the N-terminal peptide of AmyE covalently linked with a diacylglyceryl moiety (+576 Da assuming 1 C16∶0 and 1 C18∶1; estimated mass accuracy <20 ppm). The mass difference between the diacylglyceryl and the triacyl N-terminal peptide is 238 Da, which corresponds to a palmitoyl moiety. This result suggests that AmyE is diacylated in Δ*ppm2* but contains an additional modification with a palmitic acid in the wild-type and complemented strains. The absence of the *m/z* 3858.98 peak in the wild type spectrum suggests that, under these conditions, AmyE is fully tri-acylated. This was consistent with the observation that the N-terminus of AmyE purified from the wild-type strain was not accessible for sequencing by Edman degradation (data not shown). As previously shown for MSMEG_3860 (Ms-Ppm2), Cg-Ppm2 exhibits Lnt activity that results in N-palmitoylation of lipoproteins such as AmyE.

The genetic proximity between Cg-*ppm1* and Cg-*ppm2* and the fact that both corresponding proteins are encoded by the same ORF in *M. tuberculosis*, suggests that Cg-Ppm1 could be involved in lipoprotein biosynthesis in *C. glutamicum*. In order to investigate this possibility, we analyzed modifications of AmyE from a Δ*ppm1* mutant by MALDI PMF. Results showed no difference in the acylation status of AmyE between the wild type strain and the Δ*ppm1* derivative (data not shown).

### Cg-Ppm1 is Essential for Glycosylation of LppX in *C. glutamicum*

**Figure 3:**
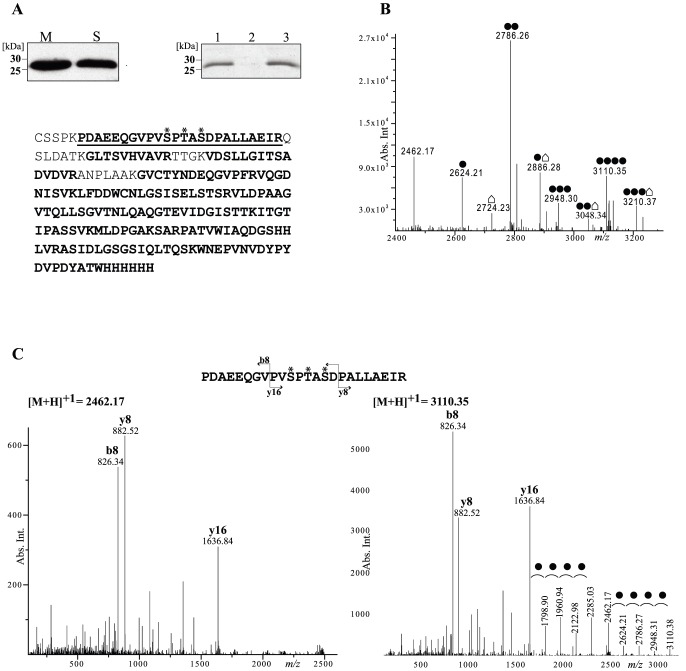
*M. tuberculosis* LppX is O-glycosylated in *C. glutamicum.* **A.** Localization of LppX in *C. glutamicum* wild-type strain: Membrane (M) and secreted (S) proteins were analyzed by SDS-PAGE, followed by immunoblotting using monoclonal anti-his antibodies (left). Purification of LppX: LppX was purified and analyzed by SDS PAGE before (lane 1) or after Triton X114 extraction. Proteins from both aqueous (lane 2) and detergent (lane 3) phases were precipitated and loaded on the gel (right). LC-ESI-MS/MS analysis of LppX peptides: Identified peptides generated by standard extraction procedures are shown in boldface type on the amino acid sequence of recombinant LppX. * indicates hydroxyl amino acid residues of the LppX_6–29_ peptide. **B.** LC-MS analysis of LppX_6–29_ glycopeptides. The deconvoluted LC-MS chromatogram is shown (21.5<RT<23.5 minutes). [M+H]^+1^ ions corresponding to the unmodified peptide (*m/z* 2462.17), glycosylated forms with 1 to 4 hexose units (*m/z* 2624.21, 2786.26, 2948.30, 3110.35) and Δ262- glycosylated forms with 0 to 3 hexose units (*m/z* 2724.23, 2886.28, 3048.34, 3210.37) are shown. • = 1 hexose (Δm = 162). • = unknown modification (Δm = 262). **C.** Deconvoluted MS/MS spectra of unmodified and tetraglycosylated peptides. Fragmentation patterns of the triply charged ions corresponding to unmodified (left) and tetraglycosylated (right) LppX_6–29_ peptides are shown. b8, y8 and y16 most intense fragment ions are annotated in both MS/MS spectra. Fragmentation pattern of the tetraglycosylated LppX_6–29_ peptidereveals 4 neutral losses of 162 Da coming from the intact tetraglycosylated peptide (*m/z* 3110.38) and from the tetraglycosylated y16 fragment ion (*m/z* 2285.03). • = 1 hexose (Δm = 162 Da).

Ppm1 proteins from *C. glutamicum*, *M. smegmatis* and *M. tuberculosis* have been characterized as PPM synthases, enzymes that transfer mannose (Man) moieties on polyprenol monophosphate from GDP-Man to produce PPM molecules that constitute Man-donors for lipoglycan biosynthesis [28], [30]. Glycoproteomics studies based on ConA lectin affinity capture of mannosylated proteins revealed that 41 proteins of *M. tuberculosis* are potentially glycosylated [14]. Among these, 18 were lipoproteins, suggesting a possible connection between protein glycosylation and acylation pathways in Mycobacteria. Tschumi *et al*., recently described the N-acylation of lipoprotein LppX in *M. smegmatis* [27]. Because LppX was also one of the proteins found to be putatively glycosylated in the glycoproteomic study [14], we sought to investigate the role of Cg-Ppm1 in post-translational modification of LppX. The DNA sequence encoding LppX-HA-His (LppX with C-terminal hemagglutinin and 6xHis epitopes [27]) was amplified by PCR from pMV261-Gm-FusLppX-HA-His and cloned under the *amyE* promoter in the shuttle *E. coli*/*C. glutamicum* vector pCGL482. When expressed in wild-type *C. glutamicum*, LppX was mainly found associated with the membrane fraction (Fig. 3A). LppX was purified by affinity chromatography from the membranes, in-gel digested with trypsin, andthe peptide mixture was analyzed by liquid chromatography-electrospray-ionization-tandem mass spectrometry (LC-ESI-MS/MS). The MS/MS data were analyzed with the Mascot Error Tolerant Search software against the recombinant LppX protein sequence. Protein coverage was 90% (Fig. 3A) and the N-terminal _6_PDAEEQGVPVSPTASDPALLAEIR_29_ peptide was found to be heterogenously glycosylated with hexose units. Accurate analysis of the MS data after deconvolution within 21.5 and 23.5 minutes of retention time revealed 1 [M+H]^+1^ molecular ion corresponding to the unmodified LppX_6–29_ (*m/z* 2462.17) and 4 [M+H]^+1^ molecular ions differing by 162 Da which correspond to the LppX_6–29_ glycopeptides with 1, 2, 3 and 4 hexose units (*m/z*2624.21, 2786.26, 2948.30, 3110.35). Curiously enough, we also observed 4 [M+H]^+1^ molecular ions corresponding to the unmodified, mono, di and tri-glycosylated LppX_6–29_ peptides with an additional single modification of 262 Da (*m/z*2724.23, 2886.28, 3048.34, 3210.37) (Fig. 3B). This modification is, however, still unknown. MS/MS data of LppX_6–29_ glycopeptides were analyzed and neutral losses of 162 Da were observed in all the MS/MS spectra of glycopeptides. This well known property is diagnostic of dissociation of hexose units from singly charged glycopeptides [34]. The MS/MS spectra of the unmodified and tetraglycosylated LppX_6–29_ peptide produced intense b8, y8 and y16 ions that were systematically detected in the MS/MS spectra of all the glycopeptides (data not shown and Fig. 3C). Hence these ions were considered as diagnostic to ascertain the amino-acidic nature of the LppX_6–29_ peptide. In the MS/MS spectrum of the tetraglycosylated LppX_6–29_ peptide, 4 neutral losses of 162 Da were efficiently observed, generated from the intact glycopeptide as well as from its y16 ion (Fig. 3C, right panel).

Together, these results show that the LppX_6–29_ peptide is highly and heterogeneously O-glycosylated in *C. glutamicum.* Similar LC-MS/MS analyses were performed with LppX protein purified from the Δ*ppm1* mutant andcomplementedstrain Δ*ppm1* (pCg-*ppm1*). While the unmodified LppX_6–29_ peptide was efficiently detected in both strains, LppX glycopeptides were only observed in the complementedstrain (data not shown). These data demonstrate that Cg-Ppm1 is absolutely required for LppX glycosylation in *C. glutamicum.*

### The N-terminal LppX_1–29_ Peptide is Acylated and Glycosylated

**Figure 4:**
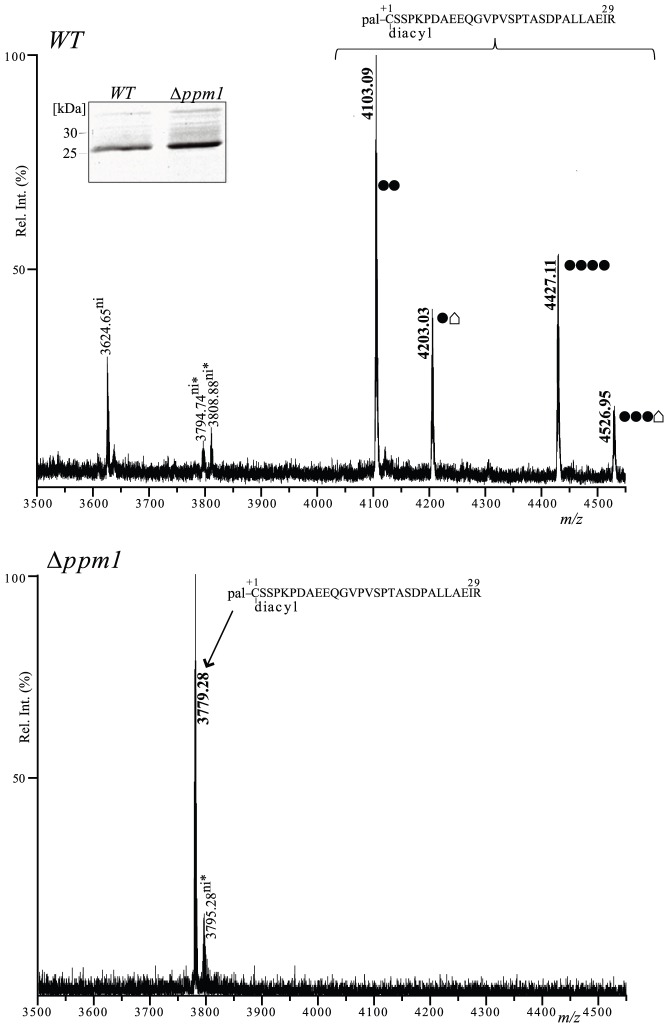
Cg-Ppm1 is required for LppX glycosylation. Comparison of MALDI PMF profiles of LppX protein purified from *C. glutamicum* wild-type and Δ*ppm1* strains. The *m/z* 3500–4550 region of the mass spectra of LppX tryptic peptides after DDM/CHCl_3_-CH_3_OH treatment is shown and significant monoisotopic [M+H]^+1^ peaks are annotated. Upper panel: *m/z* peaks correspond to glycosylated and triacylated LppX_1–29_ peptides are indicated in bold (*m/z* 4103.09, 4203.03, 4427.11 and 4526.95). Three peaks (*m/z* 3624.65, 3794.74 and 3808.88) were detected but not identified. Bottom panel: the *m/z* peak corresponding to the non-glycosylated triacylated LppX_1–29_ peptide was specifically observed in the Δ*ppm1*mutant (*m/z* 3779.28, in bold). The *m/z* 3795.28 peak was not identified. • = 1 hexose (Δm = 162 Da). • = unknown modification (Δm = 262 Da). “ni” means not identified and asterisks indicate that *m/z* assignments are not very accurate. Inset: SDS PAGE of the purified LppX proteins from the wild type and the Δ*ppm1* strains.

Purified LppX partitioned in the detergent phase when treated with Triton X-114 (Fig. 3A) suggesting that it is acylated in *C. glutamicum*. In order to evaluate the nature of this modification and its potential coexistence with glycosylation, we submitted trypsin-digested LppX to mass spectrometry analyses. The LC-ESI-MS/MS approach could not be used because triacylated peptides were not detected under standard liquid chromatography conditions (data not shown). Hence, as for AmyE, LppX peptides were extracted with dodecylmaltoside (DDM) and chloroform/methanol, then analyzed by MALDI-PMF. Two main peaks (*m/z* 4103.09 and 4427.11) were detected in the spectrum from the wild-type strain (Fig. 4A). They match to the N-terminal triacylated and glycosylated LppX_1–29_ N-terminal peptide with 2 and 4 mannose residues respectively. In the Δ*ppm1* context, these peaks were absent but a single *m/z* 3779.28 peak was detected (Fig. 4A). This peak matched to the N-terminal triacylated LppX_1–29_ peptide. All experimentally found *m/z* values are summarized and compared to the calculated *m/z* values in Table 1. The presented results clearly show that LppX N-terminus is acylated and glycosylated in *C. glutamicum* and that these modifications are not mutually exclusive.

**Table 1:** Summary of the N-terminal LppX_1–29_ peptides detected by MALDI-PMF after DDM/CHCl_3_-CH_3_OH treatment in different genetic backgrounds.

Strains	Triacylated LppX_1–29_ MH^+^ = 3778.46	Triacylated and glycosylated LppX_1–29_ (triacylated LppX + n×162)	Diacylated LppX_1–29_ MH^+^ = 3540.46	Diacylated and glycosylated LppX_1–29_ (diacylated LppX+ n×162)
WT		***4103.09 (n = 2)***		
		***4427.11 (n = 4)***		
Δ*ppm1*	**3779.28**			
Δppm1(pCg-*ppm1*)		***4103.03 (n = 2)***		
		***4427.05 (n = 4)***		
Δppm1(pMt*-ppm1*/*D1+D2*)		***4103.22 (n = 2)***		
		***4427.18 (n = 4)***		
Δ*ppm2*			3540.89	
Δppm2(pCg-*ppm2*)	**3779.23**	***3941.15 (n = 1)***		
		***4103.22 (n = 2 )***		
Δppm2(pMt*-ppm1*/*D1+D2*)	**3779.20**	***3941.22 (n = 1)***		
		***4103.26 (n = 2)***		
		***4427.28 (n = 4)***		
Δppm2(pCg-*ppm1*)				*3864.99 (n = 2)*
				*4188.82 (n = 4)*
Δppm2(pMt*-ppm1*/*D1*)				*3702.89 (n = 1)*
				*3864.87 (n = 2)*

Triacylated peptides are in **bold**. Glycosylated peptides are in *italic.* n represents the number of hexoses associated with the peptide.

Unmodified LppX _1–29_ MH^+^ = 2964.46.

### Deletion of Cg-ppm2 Affects both N-acylation and Glycosylation of LppX

**Figure 5:**
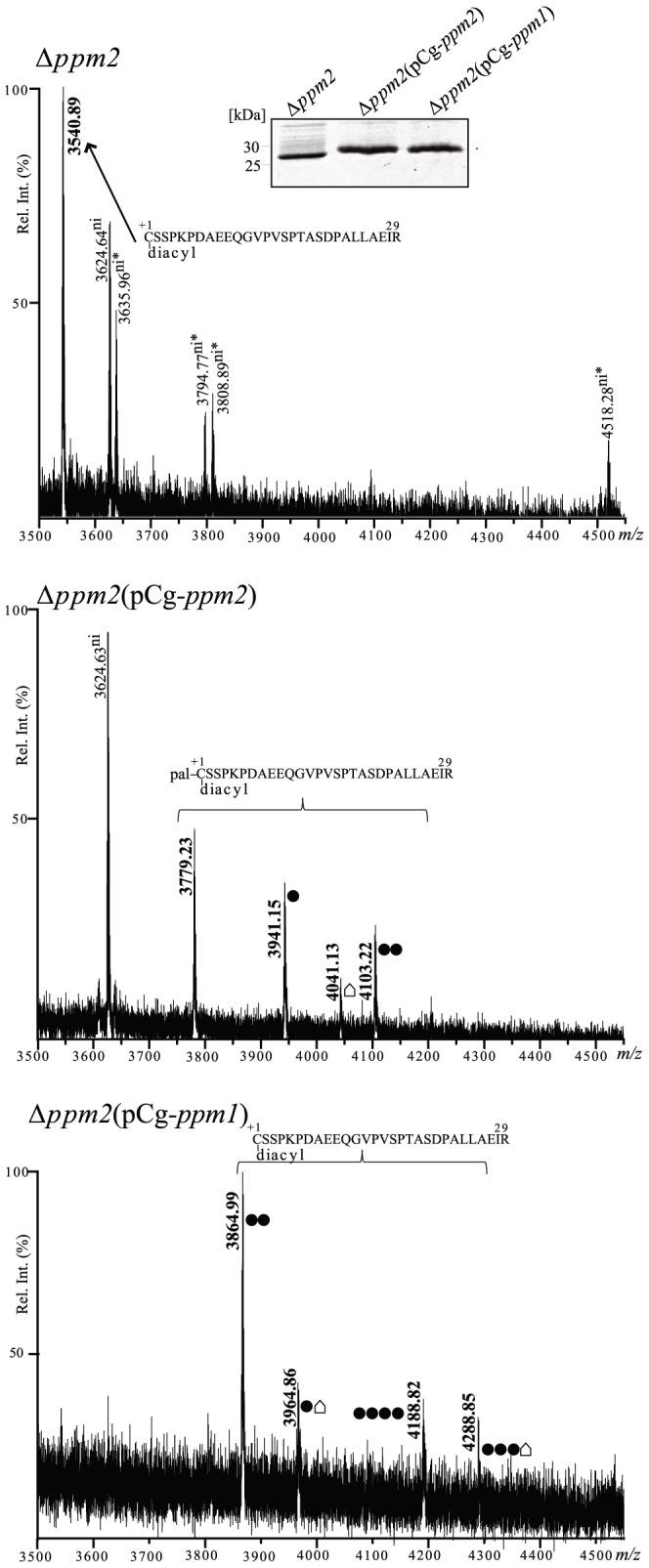
Cg-Ppm2 activity affects LppX glycosylation. Comparison of MALDI PMF profiles of LppX protein purified from ΔΔ*ppm2*, Δ*ppm2* (pCg-*ppm2*) and Δ*ppm2* (pCg-*ppm1*). The *m/z* 3500–4550 region of the mass spectra of LppX tryptic peptides after DDM/CHCl_3_-CH_3_OH treatment is shown and significant monoisotopic [M+H]^+1^ peaks are annotated. Upper spectrum: in the the Δ*ppm2* strain, only the *m/z* peak corresponding to the non-glycosylated diacylated LppX_1–29_ peptide is identified (*m/z* 3540.89, bold). Five peaks were assigned but not identified (*m/z* 3624.64, 3635.96, 3794.77, 3808.89 and 4518.28). These peaks do not match to internal tryptic LppX peptides. Middle spectrum: in the Δ*ppm2* (pCg-*ppm2*) strain, *m/z* peaks corresponding to different glycosylated forms of the triacylated LppX_1–29_ peptide are observed (*m/z* 3941.15, 4041.13 and 4103.22, in bold) as well as the peak corresponding to the non-glycosylated triacylated LppX_1–29_ peptide (*m/z* 3779.23, in bold). The *m/z* 3624.63 peak was detected but not identified. Bottom spectrum: in the Δ*ppm2* (pCg-*ppm1*) strain different glycosylated forms of the diacylated LppX_1–29_ peptide are detected (*m/z* 3864.99, 3964,86, 4188.82 and 4288.85, in bold). • = 1 hexose (ΔΔm = 162 Da). • = unknown modification (Δm = 262 Da). “ni” means not identified and asterisks indicate that *m/z* assignments are not very accurate. Inset: SDS PAGE of the purified LppX proteins from the wild type, the Δ*ppm2* and the complemented strains.

In order to test the role of Cg-Ppm2 on post-translational modifications of LppX, the protein was expressed in the Δ*ppm2* mutant. The protein, which is still associated with the membrane, was purified and analyzed exactly as the one from the wild type strain. As expected from our results on AmyE, only the diacylated N-terminal LppX_1–29_ peptide was detected (*m/z* 3540.89) in the spectrum (Fig. 5), confirming that Cg-Ppm2 has apolipoprotein N-acyltransferase activity and that it is also active on heterologously expressed mycobacterial lipoproteins. The *m/z* 3540.89 peak (diacylated peptide) is observed neither in the wild type nor in the ppm1 background suggesting that, in these conditions, LppX is fully triacylated exactly as AmyE. Interestingly, this peak corresponds to the non-glycosylated diacylated LppX_1–29_ N-terminal peptide. Moreover, only the non-glycosylated form of the LppX_6–29_ peptide was detected (data not shown), further confirming that LppX is not glycosylated when expressed in the Δ*ppm2* mutant. This could be an indication that protein glycosylation occurs downstream of protein acylation or that the absence of Cg-Ppm2 affects the stability and/or the activity of enzymes that directly contribute to lipoprotein glycosylation. The observation that post-translational modifications (triacylation and/or glycosylation) were recovered when LppX was purified from the Δ*ppm2* (pCg-*ppm2*) and the Δ*ppm2*(pCg-*ppm1*) strains (Fig. 5) favored the second functional hypothesis.

### Mt-ppm1 (Rv2051c) can Complement *C. glutamicum* Δppm1and Δppm2 Mutants

**Figure 6:**
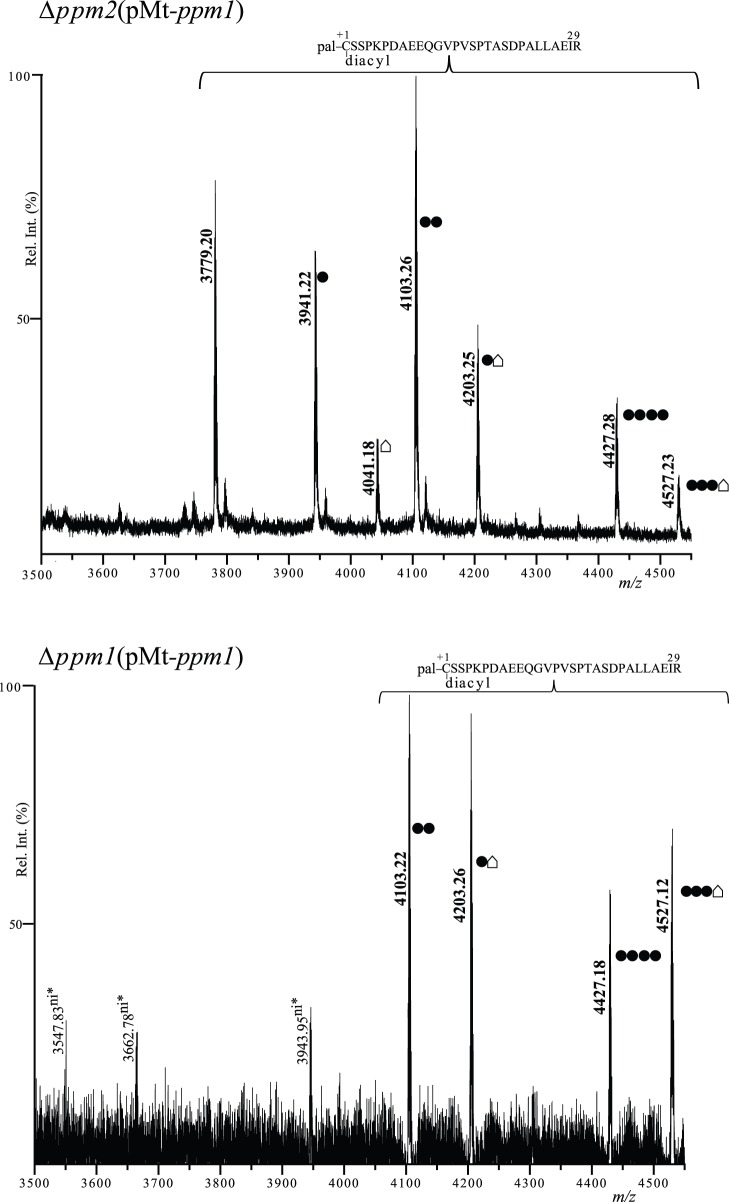
*M. tuberculosis* Ppm1 is active in *C. glutamicum.* Comparison of MALDI PMF profiles of LppX protein purified from Δ*ppm2* (pMt-*ppm1*) and Δ*ppm1* (pMt-*ppm1*). The *m/z* 3500–4550 region of the mass spectra of LppX tryptic peptides after DDM/CHCl_3_-CH_3_OH treatment is shown and significant monoisotopic [M+H]^+1^ peaks are indicated. Upper spectrum: in the Δ*ppm2* (pMt-*ppm1*) *m/z* peaks corresponding to different glycosylated forms of the triacylated LppX_1–29_ peptide were observed (*m/z* 3941.22, 4041.18, 4103.26, 4203.25, 4427.28 and 4527.23) as well as the peak of the non-glycosylated triacylated LppX_1–29_ peptide (*m/z* 3779.20) Bottom spectrum: in the Δ*ppm1* (pMt-*ppm1*) strain, peaks corresponding to different glycosylated forms of the triacylated LppX_1–29_ peptide were observed (*m/z* 4103.22, 4203.26, 4427.18 and 4527.12, in bold). Three peaks (*m/z* 3547.83, 3662.78 and 3943.95) were detected but not identified. • = 1 hexose (Δm = 162 Da). • = unknown modification (Δm = 262 Da). “ni” means not identified and asterisks indicate that *m/z* assignments are not very accurate.

LppX is triacylated but not glycosylated in a Δ*ppm1*mutant and only diacylated in a Δ*ppm2*mutant. Mt-Ppm1 exhibits polyprenol-monophosphomannose activity and it is also able to complement Ms-Ppm2 activity in *M. smegmatis* [27]. Here, Mt-*ppm1* was introduced *in trans* in *C. glutamicum*Δ*ppm1* and Δ*ppm2* mutants. LppX purified from these strains indicated restoration of both N-palmitoylation and mannosylation (Fig. 6). This result indicates that both apolipoprotein N-acyltransferase (Lnt) and polyprenol-monophosphomannose (PPM) synthase activities of Mt-Ppm1 are functional in *C. glutamicum*. Interestingly, the N-terminal domain alone of Mt-Ppm1(D1) that contains Lnt activity was not able to restore N-acylation of LppX, but restored glycosylation (Table 1). This result confirms that glycosylation does not require N-acylation and suggestsa functional interplay between protein N-acylation and glycosylation in *C. glutamicum*.

## Discussion

### Lnt Activity is Present in *C. glutamicum*

In Gram negative bacteria, the lipoprotein biosynthesis pathway is well understood and consists in a three-step post-translational mechanism that successively involves the enzymes Lgt, LspA and Lnt. Additional determinants are required for the transport of lipoproteins to the outer membrane. In the *Corynebacterineae*, only a few lipoproteins have been functionally characterized and little is known about their biosynthesis and localization. This is of medical importance since lipoproteins have been shown to contribute to virulence in *M. tuberculosis*. Lnt homologues are present in Mycobacteria, Corynebacteria and Streptomyces species. In *M. tuberculosis*, Mt-Ppm1 (Rv2051c) is a bi-domain enzyme with a N-terminal Lnt domain and a C-terminal PPM synthase domain. Interestingly, these two domains are encoded by individual adjacent ORF – named *ppm1* and *ppm2* – in the genomes of other Mycobacteria and Corynebacteria. A detailed study demonstrated that Ms-Ppm2 (MSMEG_3860) and Mt-Ppm1 (Rv 2051c) exhibit Lnt activity in *M. smegmatis* and *M. tuberculosis*, respectively, generating triacylated lipoproteins [27] The function of Ms-Ppm1 and the D2 domain of Mt-Ppm1 are supposed to be essential for lipoglycan synthesis but their role in lipoprotein biosynthesis has never been tested so far. In *C. glutamicum*, the ppm operon has been described to be essential for the synthesis of lipoglycans. While the Δ*ppm1* mutant displayed altered growth rate, inability to synthesize PPM molecules and lipoglycans, the Δ*ppm2* has a lower content of lipoglycans but no growth defect. Here, we studied the role of *ppm1* and *ppm2* of *C. glutamicum* in the post-translational modification of lipoproteins. For this purpose, we first focused on AmyE, a predicted lipoprotein, highly expressed in *C. glutamicum*. We report that AmyE is indeed a lipoprotein, post-translationally modified on its C+1 cysteine and anchored in the membrane. Experiments of membrane separation on sucrose gradients further indicated that AmyE was primarily present in the inner membrane fractions (data not shown), consistent with its function of SBP of an ABC transporter. As expected, AmyE^C1L^ is no longer post translationnally modified and therefore not associated with the membrane. Instead, AmyE^C1L^ is released from intact cells in the culture medium. This localization is surprising and suggests that the mycomembrane integrity has been compromised by the overexpression of this non-lipidated variant. Both the cell-bound wild-type and the released non-lipidated forms of AmyE were of the same size and cleaved off their signal sequence, indicating that Lsp of *C. glutamicum* is active on preprolipoproteins. This behaviour is similar to that of other Gram-positive bacteria, while Gram-negative Lsps are only active on diacylated prolipoproteins [35].

MALDI analysis of AmyE showed a difference between the calculated and the experimental mass of the intact protein, fully compatible with a triacylation. In addition, after trypsin cleavage, the N-terminal peptide of AmyE from the parental strain has a mass of 4097.10 Da instead of 3283.52 Da expected for the unmodified peptide, while the same N-terminal peptide from the isogenic Δ*ppm2* showed a mass of 3858.98 Da. After calculating all theoretical combinations of the main fatty acids found in *C. glutamicum*, we inferred that our results are a strong indication of the addition of a diacylglyceryl moiety (C16∶0 and C18∶1) completed by an N-palmitoylation (C16∶0). In total, the observed modification accounts for 814 Da. We found exactly the same modifications on *M. tuberculosis* LppX expressed in *C. glutamicum*. This result indicates that lipoprotein-modifying enzymes (including Lgt and Lnt) of *C. glutamicum* are active on heterologously expressed mycobacterial lipoproteins. In a previous report, Tschumi *et al.* found a mass difference of 831 Da due to post-translational modification of LppX expressed in *M. smegmatis* [27]. This included a diacylglycerol modification (tuberculostearic acid C19∶0 and C16∶0)and an N-palmitoylation (C16∶0). The slightly smaller value we found (814) when LppX was expressed in *C. glutamicum* is likely due to the absence of mycobacterial specific fatty acids. Indeed, tuberculostearic acid is a 10-methyloctandecanoic acid that may account for the mass difference of +17 Da observed for LppX modification in *M. smegmatis* as compared to the C18∶1 in *C. glutamicum*. The N-palmitoylation of AmyE and LppX (+238 Da) does not occur in a Δ*ppm2* mutant, confirming that Cg-*ppm2* has Lnt activity similar to that of its homolog in *M. smegmatis*. All together, our results constitute a second report that shows the presence of an Lnt activity in *Corynebacterineae* and therefore confirms that these outer membrane-containing Gram-positive bacteria are able to triacylate lipoproteins, just as Gram-negative bacteria. In Gram-negative bacteria, depletion of Lnt causes mislocalization of outer membrane proteins [36]. In *C. glutamicum*, both AmyE and LppX were anchored to the membrane in the presence or in the absence of Cg-Ppm2, indicating that protein diacylation is sufficient for membrane tethering. Because AmyE and LppX are found mainly associated to the cytoplasmic membrane (data not shown), we cannot determine a putative role of Cg-Ppm2-mediated triacylation in the export of lipoproteins to the mycomembrane. In any case, the absence of Cg-Ppm2 did not cause any defect in cell envelope stability or cell growth. The precise role of protein N-palmitoylation in the cell physiology remains to be elucidated.

### Lipoprotein Glycosylation in *C. glutamicum*

Besides acylation, LppX was found to be O-glycosylated in *C. glutamicum*. This glycosylation is attributable to hydroxyl-bearing amino acids within the peptide ^6^PDAEEQGCPVSPTASDPALLAEIR^29^. This peptide contains two serines (S16 and S20) and one threonine (T18) in proximity to prolines, a feature reminiscent of glycosylation targets in Actinomycetes [15], [16], [34], [37]. Ser 16 and Thr 18 were also predicted as potential targets for O-glycosylation by the Net-OGlyc 3.1 software (data not shown). However, we were not able to locate the exact sites of glycosylation using conventional CID MS/MS techniques because of the lability of glycosidic bonds. Since in Gram-negative bacteria amino acids at positions +2 and +3 are essential for the correct localization of protein in the inner or the outer membrane [38] it is interesting to note that the atypical double serine residues at the +2 and +3 position of LppX were not found to be glycosylated in our study. This is similar to LpqH from *M. tuberculosis*, which also contains a double serine at positions +2 and +3, but has been shown to be glycosylated somewhere in the threonine cluster, located between amino acids 13 and 20. Even though there is no sequence homology between LpqH and LppX, the position of the glycosylation sites may have been conserved in the two proteins. The glycosylation of LppX observed in *C. glutamicum*most likely reflects its modification in Mycobacteria. Indeed, in *M. smegmatis,* LppX has been shown to be post-translationally modified by hexoses (P. Sander, personal communication) and in *M. tuberculosis*, LppX has a high affinity for ConA-lectins [14]. This latter result suggests that LppX is indeed mannosylated, which is in line with our results, showing that the LppX_6–29_ glycopeptide can be deglycosylated by treatment with alpha-mannosidase (data not shown). The function of LppX glycosylation is still unknown but, might be related to its cell wall localization and could be essential for translocation of complex lipids (DIM) to the outer leaflet of the outer membrane as it was proposed for SodC [18] or LpqH [16], [39]. Finally, it is worth noting that besides mannosylation, an additional modification of 262 Da was consistently observed for the LppX_6–29_ peptide. Putatively, it could represent a mannose hydrophobic derivative, since it increases the hydrophobicity of the modified peptide (data not shown). To our knowledge, this difference of mass does not correspond to any known post-translational modification and it has been described once without molecular assignment [40].

We showed that the PPM synthase Cg-Ppm1 is required for LppX glycosylation in *C. glutamicum*. PPM synthases are ubiquitous throughout *Corynebacterineae* in which they catalyze the transfer of mannose from GDP-mannose to lipid carriers polyprenol phosphates [28], [30]. Then, cytoplasmic PPM flips the inner membrane to act as a mannose donor for the biosynthesis of LM and LAM (see Briken *et al.*, for a review [41]). The direct involvement of Ppm1 in protein glycosylation in Actinomycetes has been demonstrated recently. In *Streptomycescoelicolor*,both Ppm1 and the *O-*mannosyltransferase Pmt are part of a protein glycosylation pathway [15], [42]. This closely resembles the pathway for the biosynthesis of *O-*mannosylated proteins in eukaryotes [43], [44]. Similarly to *S. coelicolor*, Pmt of *C. glutamicum* (NCgl0854) and *M. tuberculosis* (Rv1002c) are functional orthologs of eukaryote PMTs [44], [45], [46]. Cg-Pmt is required for the glycosylation of secreted proteins including the resuscitation promoting factor 2 (Rpf2) and the putative lipoprotein LppS [45]. In this context, our results indicate that, in addition to its essential role in LAM and LM synthesis, Cg-Ppm1 is also a key element for the glycosylation of lipoproteins in *C. glutamicum*.

### Mt-Ppm1 is Active in *C. glutamicum*

In *M. tuberculosis,* Ppm1 and Ppm2 proteins are encoded by the single ORF Rv2051c (Mt- *ppm1*). Here we show that Mt-Ppm1 is able to complement the Δ*ppm2* mutant for LppX acylation and glycosylation and the Δ*ppm1* mutant for LppX glycosylation. This result indicates that Mt-Ppm1 is functional in *C. glutamicum* and does not show any host specificity. Tschumi *et al.* [27] previously reported that Ms-Ppm2 was not able to complement its *E. coli* homolog even after exchange of the two essential amino acids differing between *E. coli* and *M. smegmatis*. The authors suggested that this lack of complementation could be due to the fact that Ms-Ppm2 only recognizes lipoproteins modified with a diacylglyceryl residue carrying at least one long chain fatty acid, the tuberculostearic acid. Our data show that Mt-Ppm1is able to complement Cg-Ppm2 and is therefore able to recognize a diacylglyceryl residue without tuberculostearic acid. Hence, the absence of complementation of Ms-Ppm2 observed by Tschumi *et al.* [27] could be due to low expression level or low enzymatic activity of Ms-Ppm2, which may not sustain growth of *E. coli*, rather than to substrate specificity.

### Glycosylation of LppX is Dependent on the Presence of Cg-Ppm2

Interestingly, we found that the deletion of the Cg-*ppm2* gene caused the complete loss of glycosylation of LppX. This observation was unexpected and might be explained as follows. First, the absence of glycosylation of LppX may suggest that tri-acylation of LppX is a prerequisite for glycosylation. This hypothesis has been ruled out since overexpression of Cg-*ppm1* in a Δ*ppm2* mutant restores glycosylation and that Mt-Ppm1/D1 (N domain of Rv2051c),which is not able to restore acylation in the Δ*ppm2* mutant,is nevertheless able to restore glycosylation. Second,because Cg-*ppm1* is located immediately downstream of Cg-*ppm2,* a possible decrease in Cg-*ppm1* transcription could not be excluded and may explain our observations. LAM and LM production as well as *in vitro* activity of Cg-Ppm1 on artificial acceptors are decreased in the Δ*ppm*2 mutant as compared to the wild type strain and could not be restored by complementation (our unpublished data and [28]). This observation clearly indicates that there is indeed a polar effect of Cg-*ppm2* deletion on Cg-*ppm1* expression. However, this decrease in Cg-*ppm1* transcription, could notalone explain the absence of LppX glycosylation in the Δ*ppm2* mutant since glycosylation (and triacylation)were recovered when LppX was purified from the Δ*ppm2* complemented strain (Δ*ppm2* (pCg-*ppm2*)). Finally, a third possibility could be that Cg-Ppm2, in addition to its apolipoprotein N-acyltransferase activity, is able to interact with Cg-Ppm1 and increase its activity by anchoring the protein at the membrane surface. This hypothesis is in line with the results of Baulard *et al*. who demonstrated a direct interaction between Ms-Ppm1 and Ms-Ppm2 in *M. smegmatis* [29]. Because lipoprotein glycosylation, but not lipoglycans synthesis, can be restored by complementation of the Δ*ppm2* mutant with pCg-*ppm2*, we propose that Cg-Ppm2 could even mediate a physical interaction between Cg-Ppm1 and Pmt in order to efficiently drive the transfer of mannose directly from the phosphoprenol carrier to the lipoprotein. This interesting perspective pointing to unexpected links between lipoglycans and lipoprotein biogenesis awaits further analysis.

## Experimental Procedures

### Strains and Plasmids

All *C. glutamicum* strains used in this study are derivatives of ATCC13032 RES167 [47]and were routinely cultured on Brain Heart Infusion (BHI) medium (DIFCO) at 30°C. The construction of mutants Δ*ppm1*and Δ*ppm2* has been described elsewhere [28], *E. coli* DH5α was used as a host for cloning. *C. glutamicum* strains carrying pCGL482 [48] or pVWEx2 [49] derivativeplasmids were grown in the presence of chloramphenicol (Cm) and tetracycline (Tet) at final concentrations of 25 µg ml^−1^ and 5 µg ml^−1^ respectively. Transformations of *C. glutamicum*were performed by electroporation as described by Bonamy *et al.* [50]. Primers synthesis and DNA sequencing were performed by Eurofins MWG operon.

### Construction of Expression Vectors

The DNA region encoding AmyE-6his was amplified by PCR using genomic DNA from *C. glutamicum* ATCC 13032 as a template, the forward primer (5′-AGCGGA*GGATCC*TGTATTTGTATGTTTTAGGCCCG-3′) with a *BamHI* site and the reverse primer (5′-CGT*CTCGAG*TTAATGATGATGATGATGATGGCCCCAGTTGGATTCC-3′) with a *XhoI* site. The reverse primer also contained a sequence encoding an in frame C-terminus hexahistidine tag (underlined). The resulting PCR product – *amyE-his* with a 200 bp upstream region – was digested with *Xho*I and *BamH*I and ligated into the same sites of pCGL482, generating pCGL482-AmyE. Expression of *amyE* from this plasmid was driven by its own native promoter.

The DNA region encoding LppX-HA-His was amplified by PCR from plasmid pMV261-Gm-Fus-LppX-HA-His [27] with the forward primer (5′-CGTAGTC*CTCGAG*AAGCTTGCATGCCTGC-3′) with a *XhoI* site and the reverse primer (5′-CGT*GATATC*ACGACAGGCAAAGGAGCACAGGATGAATGATGG-3′) with an *EcoRV* site. After digestion with *EcoRV* and *XhoI*, the PCR product was inserted into appropriately restricted pCGL482-AmyE, resulting in pCGL482-LppX, in which expression of *lppX* is under the control of the *amyE* promoter.

### Construction of Plasmids Expressing ppm Genes

Cg-*ppm1* was cloned under the tac promoter in pVWEx2 as described previously [28]. Cg-*ppm2*, Mt-*ppm1*/D1 and Mt-*ppm1*/D1+D2 were similarly cloned in the same vector by using the following primers:

Cgppm2_XbaI_F CAT GCA TG*TCTA GA*A AGG AGA TAT AGA TAT GAC ACT GTT TGT TCG GCT C, Cgppm2_BamHI_R CAT GCA TG*G GAT CC*T TAT TTT ACT TTT CGA CGA TT, Mtppm1D1_XbaI_F CAT GCA TG*TCTA GA*A AGG AGA TAT AGA TAT GAA GCT TGG CGC CTG GGT G, Mtppm1D1_BamHI_R CAT GCA TG*GGAT CC*T TAC ATG TAA CTC CTC GAT GT, Mtppm1D1_XbaI_F CAT GCA TG*TCTA GA*A AGG AGA TAT AGA TAT GAA GCT TGG CGC CTG GGT G and MtppmD2_XbaI_R GAT C*TC TAG A*TC ATT CGG TCA CGT CGG CGC GGC.

### Site-directed Mutagenesis

Site-directed mutagenesis of the *amyE* gene was performed with a QuickChange XL mutagenesis kit (Stratagene) according to the manufacturer’s protocol.In order to replace the cysteine +1 by a leucine in the ORF of AmyE, we performed a PCR reaction on pCGL482-AmyE with primers Cys/Leu1 (5′GTC GGT GGA GCC GGA CAACGC GAC GAG ACT TGC 3′) and Cys/Leu2 (5′GCA AGT CTC GTC GCG TTG TCC GGC TCC ACC GAC 3′). The resulting product was treated with *Dpn*I to degrade the parental plasmid and directly transformed into DH5α *E. coli* cells. The presence of the mutation was confirmed by sequencing pCGL482-AmyEC1L with the primer 482S (5′ GCAGAATAAATGATCCGTCG 3′) and was introduced in *C. glutamicum* strain ATCC 13032.

### Cell Fractionation

10 ml cultures of *C. glutamicum* were grown overnight in BHI medium at 30°C with vigorous shaking (200 rpm). 1 ml culture aliquots were centrifuged, cell pellets were resuspended 1 ml of 50 mM Tris-HCl pH 7.0 with glass beads and vortexed for 15 min. Unbroken cells were discarded and total membranes were collected by centrifugation (65,000×g, 30 min.) in a Beckman TLA 100.3 rotor. Proteins in the supernatant were precipitated with 10% trichloroacetic acid (TCA) at 4°C for 30 min. The precipitated proteins were recovered by centrifugation (13,000×g; 15 min) and washed with cold acetone. Protein samples were analyzed on SDS-PAGE and immunoblotting.

### Purification of 6His-tagged Proteins

6His-tagged AmyE and LppX proteins were expressed in wild-type *C. glutamicum*, Δ*ppm1* and Δ*ppm2* mutants and purified from their respective membrane fractions. 300 ml of BHI was inoculated with the appropriated strain and incubated at 30°C with vigorous shaking (200 rpm) overnight. Cell pellets were collected by centrifugation, washed and resuspended in 10 ml of 25 mM HEPES pH 7.4. Cells were broken by three passages through a French pressure cell at 18,000 psi. Unbroken cells were removed by low-speed centrifugation (4,000×g for 15 min) and the cleared supernatant was then centrifuged at 65,000×g for 30 min (rotor Beckman TLA 100.3). Total membranes were homogenized in 25 mM phosphate buffer pH 8.0 containing 0.1 mg/ml Pefabloc and 4% LDAO (lauryl-dimethylamine-oxide), and stirred for 30 min room temperature. Insoluble material was removed by centrifugation at 65,000×g for 30 min. Solubilized membrane protein extracts were saved and loaded on a Ni-NTA column (Quiagen Ni-NTA superflow) previously equilibrated with 25 mM phosphate buffer pH 8.0, 2% LDAO (buffer A). The column was washed with ten column volume of buffer A containing 10 mM imidazole and His-tagged proteins were eluted with buffer A containing 250 mM of imidazole. AmyE^C1L^ was concentrated from a 300 ml-cell culture supernatant using ultrafiltration units (Amicon YM 10 kDa) (1 ml/min for 5 h at 4°C). The resulting protein concentrate was dialyzed extensively against 25 mM phosphate buffer pH 8.0 at 4°C and loaded on a Ni-NTA column and purified as described. Elution fractions were analyzed by SDS-PAGE and Coomassie Brilliant Blue staining. Purified proteins were dialyzed against buffer A and stored at −20°C until use.

### Triton X114 Extraction

Purified proteins were diluted 10-fold in 25 mM HEPES buffer pH 7.4 containing 2% Triton X-114. Lipoprotein extraction was performed at 4°C for 2 h 30 min. on a rocking platform. After incubation at 37°C for 10 min and centrifugation at 12, 000×g for 10 min., the detergent phase and the aqueous phase were separated. Proteins in both phases wereTCA-precipitated as described, resuspended in Laemmli buffer and analyzed by SDS-PAGE and immunoblotting.

### Mass Spectrometry Analysis

#### MALDI (matrix-assisted laser desorption/ionisation) protein mass measurements

Purified recombinant proteins (0.5 µl) were mixed with an equal volume of the sinapic acid matrix (Sigma-Aldrich; 10 mg/ml, 50% CH_3_CN, 1% trifluoroacetic acid (TFA)). Crystals were obtained using the dried droplet method, and ∼300 mass spectra were averaged per spot. Acquisitions were performed in positive linear mode with the Voyager-DE STRMALDI-TOF mass spectrometer (ABSCIEX). External calibration was performed with enolase (M = 46672 Da) and bovine serum albumin (M = 66431 Da) of the promix 3 mixture (LaserBio Labs).

#### MALDI Peptide Mass Fingerprinting (PMF)

Enzymatic digestion of excised bands was performed using the Progest robot (Genomic Solutions). Briefly, protein bands were extensively washed with CH_3_CN and 25 mM NH_4_HCO_3_. The excised bands were treated with 100 µl 10 mM DTT at 57°C for 30 min. After DTT removal, 100 µl of 55 mM iodoacetamide was added for cysteine carbamidomethylation, and the reaction was left to proceed at room temperature for 30 min. After removal of the supernatant, the washing procedure was repeated, and then gel slices were dried. Twenty microliters of 10 ng/µl Porcine Gold Trypsin (Promega) diluted in 25 mM NH_4_HCO_3_ was added, and enzymatic digestion was performed overnight at room temperature. Peptides were extracted either with standard procedures (40%H_2_O, 60% CH_3_CN, 0.1%TFA) or following a protocol described by Ujihara [33] with minor modifications. 20 µl of 1% dodecyl maltoside (Anatrace, n-Dodecyl-β-D-Maltopyranoside DDM, Sol-Grade) was added and the solution was mixed (1500 rpm) for 1 hour at room temperature; 35 µl of a 2∶1 chloroform:methanol solution (CHCl_3_-CH_3_OH) was added and the solution mixed as before. Peptide extracts from the aqueous and organic phases (0.5 µl) were quickly spotted onto a 0.5 µl droplet of 2,5-dihydroxybenzoic acid (Sigma-Aldrich; 20 mg/ml, 50% CH_3_CN, 0.1%TFA). Peptide mixtures were analyzed in positive reflectron mode with the Voyager-DE STRMALDI-TOF mass spectrometer (ABSCIEX) and ∼500 mass spectra were averaged per spot. Mass spectral analyses were performed with the Data Explorer software (ABSCIEX). MALDI PMF spectra were internally calibrated with tryptic peptides of recombinant proteins: *m/z* 3451.65 and 4045.88 for AmyE in *C. glutamicum* wild-type, Δ*ppm2* and Δ*ppm2*(pCg-*ppm2*) strains. *m/z* 2760.30 and 3451.65 for AmyE^C1L^ in the wild-type strain. *m/z*3296.76 *and m/z* 2211.10(autolytic peptide of trypsin) forLppX in wild-type, Δ*ppm2*, Δ*ppm2*(pCg-*ppm2*), Δ*ppm2*(pCg-*ppm1*), Δ*ppm2*(Mt-*ppm1*) and Δ*ppm1*(Mt-*ppm1*) strains. *m/z* 2462.24 and 3296.76 for LppX in Δ*ppm1*strain.

#### NanoLC-ESI-MS/MS analyses

Trypsin-generated peptide mixtures extracted with standard procedures (see above) were dried under vacuum, then resuspended in aqueous solution (0.1% HCOOH) and finally analyzed with the Q/TOF Premier mass spectrometer (Waters) coupled to the nanoRSLC chromatography (Dionex) equipped with a trap column (Acclaim PepMap100 C18, 75 µm I.D.×2 cm, 3 µm, nanoViper) and an analytical column (Acclaim PepMapRSLC C18, 75 µmI.D.×15 cm, 2 µm, 100 Å, nanoViper). The loading buffer was H_2_O/CH_3_CN/TFA (98%/2%/0.05%), buffer A and B were H_2_O/HCOOH (0.1%) and CH_3_CN/HCOOH (0.1%), respectively. A 2–50% B gradient was set for 40 minutes with a flow rate of 0.3 µl/min. Data-dependent scanning was applied to generate MS/MS spectra with a collision energy ramp of 15 to 40 volts. Standard MS/MS acquisitions were performed on the top of the three most intense parent ions of the previous MS scan. Raw data were processed with ProteinLynx Global Server (Waters). Peptide identification was achieved using the Mascot software with the following parameters: data bank recombinant LppX protein; peptide tolerance 15 ppm; fragment tolerance 0.1 Da; digest reagent trypsin (cleavage allowed even before proline); variable modification oxidation of methionine; fixed modification carbamidomethylation of cysteines. MS and MS/MS spectra of glycosylated peptides were processed and analyzed using the MassLynxV4.1 and the PepSeq software (Waters).

#### Mannosidase treatment

LppX tryptic peptides extracted with standard procedures, were dried and resuspended in 8 µl of 25 mM NH_4_HCO_3_. 2 µl (10 µg) of Jack bean α-Mannosidase solution (Sigma-Aldrich) was added and the reaction was left to proceed for 3 h at 37°C, while shaking (1500 rpm). Mannosidase-treated peptides (0.5 µl) were mixed with an equal volume of DHB matrix solution (Sigma-Aldrich; 20 mg/ml, 50% CH_3_CN, 0.1%TFA) and spotted using the dried-droplet method. MALDI-PMF acquisitions and data analyses were performed as described above.

### Standard Biochemical Analysis

For Western blotting, protein samples were analyzed on 12% SDS-PAGE minigels and transferred for 1 h at 30 V onto nitrocellulose membrane (Whatman Protran BA85 0.45 µm). The membranes were probed with horseradish peroxidase-conjugated anti-His antibodies diluted at 1/3000(Roche). Western Lightning® Plus–ECL (Perkin Elmer) was used for detection.

For N-terminal sequencing, proteins were separated by 12% SDS-PAGE, blotted onto a PVDF membrane and stained with Amido Black (0.1% (w/v) Amidoblack, 40% (v/v) methanol, 1% (v/v) acetic acid) for 10 min. The membrane was washed with distilled water. Visible bands were cut out and analyzed by Edman degradation at the Proteomics Platform of the Institut Pasteur Paris.
